# Identification of protein inhibitor of activated STAT 4, a novel host interacting partner that involved in bovine viral diarrhea virus growth

**DOI:** 10.1186/s12985-020-01330-0

**Published:** 2020-04-22

**Authors:** Xiaowei Gong, Qiwei Chen, Fuying Zheng

**Affiliations:** State Key Laboratory of Veterinary Etiological Biology, Lanzhou Veterinary Research Institute, Chinese Academy of Agricultural Sciences, No. 1 Xujiaping, Yanchangbao, Lanzhou, 730046 China

**Keywords:** BVDV C protein, Yeast two-hybrid, PIAS4, RNA interference, Co-immunoprecipitation

## Abstract

**Background:**

Bovine viral diarrhea virus (BVDV) belongs to the *Flaviviridae* family and the *pestivius* virus group. BVDV is responsible for significant economic loss in cattle industry worldwide because of reducing reproductive performance, increasing incidence of other diseases and mortality among young stock. The core (C) protein of the *Flaviviridae* family member is involved in host antiviral immune response through activation of related signaling pathways that affect the viral replication. However, the influence of C protein-interaction partners in BVDV infections is poorly defined.

**Methods:**

To explore C-protein-interacting partners, yeast two-hybrid was used to screen the interaction protein of C protein using bovine peripheral blood mononuclear cell (PBMC) cDNA library. The co-immunoprecipitation and confocal assays were manipulated to determine the interaction between potential partners and C protein. Knockdown and overexpression of the partner were used to examine whether the C-protein-interacting partner plays a role in BVDV proliferation and virulence. Meanwhile, qRT-PCR and western blot assays were used to investigate the effect of C protein and C-protein-interacting partner on the immune response of host cells.

**Results:**

We identified protein inhibitor of activated STAT 4 (PIAS4) as a novel interacting partner of the BVDV C protein. Co-immunoprecipitation and confocal assays demonstrated a strong interaction between C protein and PIAS4. Silencing of PIAS4 with small interfering RNA suppressed C protein expression and BVDV growth, while overexpression of PISA4 increased C protein expression and BVDV growth. The overexpression of PIAS4 increased the cell apoptosis. Meanwhile, the expressions of STAT4, SOCS3, IFITM, IFN-α were negatively regulated by the expression of PIAS4. The expression of C protein suppressed the antiviral proteins expression, and the inhibition effect was enhanced by interaction of PIAS4 and C protein. These results highlighted the beneficial properties of cellular PIAS4 for BVDV protein expression and growth.

**Conclusions:**

This study provides reliable clues for understanding the roles of PIAS4 in the regulation of BVDV growth.

## Background

Bovine viral diarrhea (BVD) can be an acute or chronic disease of cattle and is characterized by reproductive disorders, enteritis, mucosal disease, persistent infection and immunosuppression [1]. The disease is caused by bovine viral diarrhea virus (BVDV) belonging to the *pestivirus* genus within the *Flavivirus* family [2]. BVDV has a single stranded positive sense RNA genome, approximately 12.3 Kb in length, and consists of a single open reading frame (ORF) flanked by 5′ and 3′ untranslated regions (UTR). ORF is translated as a polyprotein, and the order of the individual viral proteins is as follows: N^pro^-Core (C)-E^rns^-E1-E2-P7-NS2/3-NS4a-NS4b-NS5a-NS5b [3]. The proteins associated with the mature virion are glycoproteins E^rns^, E1, E2 and the C protein.

Previous studies on host-virus interaction of BVDV were mainly focused on structural protein E and non-structural protein N. BVDV N^pro^ protein mediated the BVDV induced immunosuppression through interaction with cellular S100A9 protein [4]. BVDV N^pro^ protein and structural protein E played important roles in inhibiting type I interferon [5]. But the information on C protein-interacting proteins and their impact on BVDV infection is limited. Studies on C protein of other pestivirus species have demonstrated some important functions of C protein [6].

C protein is the virion nucleocapsid protein, which is highly basic and relatively conserved among different pestivirus species. C protein was shown to influence the regulation of cellular transcription and interact with cellular SUMOylation pathway in the classical swine fever virus growth cycle [7, 8]. In addition, C protein is dispensable for virus propagation [6, 9], and can bind cellular IQGAP1 protein, influencing CSFV virulence [9]. However, the C protein of BVDV has been characterized as lacking significant secondary structures and binds RNA with low affinity and specificity [2]. Recent studies has shown C protein to be associated with a range of cellular proteins involved in cellular signaling pathways [10], and regulated the cellular transcription, and pathogenesis [6, 11, 12]. To date, few studies have demonstrated the role of C protein of CSFV in virus replication and virulence [12, 13]. So, there is no doubt that core protein may plays a vital role in BVDV infection.

The objective of this study was to identify host proteins that directly interact with BVDV C protein, and to elucidate of the role of C protein and host protein during BVDV infection.

## Materials and methods

### Yeast two-hybrid (Y2H) screen

The matchmaker gold yeast two-hybrid system (Clontech) was used for this study. The bait plasmid pGBKT7-Core (Table 1) was transformed into yeast strain Y2H and expressed as a fusion with the Gal4 DNA-BD. The bovine peripheral blood mononuclear cell (PBMC) cDNA library (Takara), which expressed fusions with the Gal4 AD, was provided in yeast strain Y187. When cultures of the two transformed strains were hybridized overnight, they mated to create diploids. Diploid cells were identified by grow on different culture mediums, in order, double dropout medium SD/−Leu/−Trp (DDO), quadruple dropout medium SD/−Ade/−His/−Leu/−Trp (QDO) and QDO supplemented with X-α-Gal and Aureobasidin A (QDO/X/A). Plasmids from blue colonies were rescued, and targeted insertions were sequenced. The NCBI BLAST program was used to identify the host proteins. To confirm the interaction between C protein and host proteins, a control mating was performed when bait screening the library. pGBKT7–53 and pGADT7-T was performed as a positive control and a negative control should also be performed using pGBKT7-Lam and pGADT7-T. For positive interactions, both plasmids can activate all four reporters, and the numbers of blue colonies should grow on QDO/X/A, but for negative, no colonies grow on QDO and QDO/X/A. To further confirm the interaction between core and host protein, the bait plasmid (pGBKT7-Core) and pre plasmid (pGADT7-PIAS4) (Table 1) were transformed into the yeast strain Y2H and Y187, respectively.

**Table 1:** Primers used in this study

Primer	Sequence(5′-3′)	Usage
BD-C-F	CCG***CATATG***TCCGACACAAATGCAGAAGGGGC	Amplification of C Protein
BD-C-R	GCG***GAATTC***TTATCCCACTGCAACCTGAA	Amplification of C Protein
HA-C-F	CG***GAATTC***GCTCCGACACAAATGCAGAA	Amplification of C Protein
HA-C-R	GG***GGTACC***TTATCCCACTGCAACCTGAAAC	Amplification of C Protein
Myc-PIAS4-F	TAT***CTCGA*G**CGATGGCGGCGGAACT	Amplification of PIAS4
Myc-PIAS4-R	TAT***GGATCC***GCAGGCCGACACCAGA	Amplification of PIAS4
AD-PIAS4-F	TT***CATATG***GCGGCGGAACTGGTGGAG	Amplification of PIAS4
AD-PIAS4-R	ATA***CTCGAG***TCAGCAGGCCGACACCAGAC	Amplification of PIAS4
ISG15-F	GCAGACCAGTTCTGGCTGTCT	Amplification of ISG15
ISG15-R	CCAGCGGGTGCTCATCAT	Amplification of ISG15
MX1-F	GAGGTGGACCCCCAAGGA	Amplification of MX1
MX1-R	CCACCAGATCGGGCTTTGT	Amplification of MX1
OAS1-F	CCAAGTCAAACAAGCCATCGA	Amplification of OAS1
OAS1-R	CACATCGGAAACACCTCTCCTT	Amplification of OAS1
IFITM3-F	CGTGTGGTCCCTGTTCAAC	Amplification of IFITM3
IFITM3-R	CCATCTTCCGGTCCCTAGAC	Amplification of IFITM3
GAPDH-F	AAGGCCATCACCATCTTCCA	Amplification of GAPDH
GAPDH-R	CCACCACATACTCAGCACCAGCAT	Amplification of GAPDH

### Plasmids

The pCDNA3.1-PIAS4-Myc plasmid encoding the PIAS4 protein (GenBank accession no. NM_001083482) fused to a Myc tag at its N terminus was constructed by cloning PIAS4 cDNA into pCDNA3.1/myc-His (−) A vector with the Xho I and BamH I restriction enzymes. The BVDV C protein cDNA was then cloned into the pCMV-HA vector, using EcoR I and Kpn I restriction enzymes, to designated pCMV-HA Core. All plasmid sequences were verified by sequencing and amplification (Table 1).

### Cell, viruses and virus titration assays

HEK293T, MDBK, BHK21 cells were maintained in Dulbecco’s modified Eagle’s medium (DMEM), and supplemented with 10% BVDV-free fetal bovine serum (FBS), 100 U/ml penicillin, and 100 μg/ml streptomycin. The BVDV strain OregonC24V was propagated in MDBK cells. Virus titers in the culture supernatants of BVDV-infected MDBK cells were determined by the Reed-Muench method [14].

### Transfection of plasmid DNA

Transfection of plasmids (4 μg each) was performed in 6-well plates (Nunc) in a humidified 37 °C CO_2_ in incubator using Lipofectamine 2000 (Invitrogen) as the manufacturer’s instructions described. At 6 h post-transfection (hpt), the supernatants were removed and replaced with complete growth medium. The plates were incubated for an additional 48 h before being used for CO-IP and confocal assays.

### Virus infection

Forty-eight hours after DNA or small interfering RNA (siRNA) transfection, cell were infected with the BVDV strain Oregon C24V at a multiplicity of infection (MOI) of 1. Two hours later, the viral inoculum was removed and the washed twice with phosphate-buffered saline (PBS) (PH = 7.4) and then refeed with DMEM containing 2% FBS. 48 h later, cell free culture supernatants and cell lysates were harvested.

### Co-immunoprecipitation (co-IP)

Co-IP of PIAS4 with C protein: HEK293T cells were co-transfected with pCDNA3.1-PIAS4-Myc and pCMV-HA plasmids, as described above. The transfected cells were harvested at 48 hpt, washed three times with cold phos-phate-buffered saline (PBS) (pH = 7.4), and lysed with cold NP-40 buffer (50 mM Tris, 150 mM NaCl, 0.5% NP-40, 0.5 mM EDTA) containing 1 mM phenylmethylsulfonyl fluoride (PMSF) and 1 mg/ml protease inhibitor cocktail (Roche) for 2 h at 4 °C. The cell extract was centrifuged and supernatant were combined with rabbit anti-Myc polyclonal antibody (PAbs) (C3956; Sigma) at 4 °C for overnight, followed by the addition of precleared protein A/G beads (A10001L; Abmart) and incubated at 4 °C for 3 h with gentle rotation. Then, the beads was washed with NP-40 buffer 3 times and boiled in sample buffer. The immuno-precipitates were separated and analyzed by SDS–PAGE. The following antibodies: mouse anti-HA (M20003; Abmart) and mouse anti-Myc (M20002; Abmart) monoclonal antibodies (MAb) were used.

Co-IP of BVDV C protein with endogenous PIAS4: The BVDV-infected (+) and mock-infected cells were suspended and lysed in lysis buffer (NP-40 buffer, 1 mM PMSF and 1 mg/ml protease inhibitor cocktail). Total cell lysates were clarified by centrifugation and supernatant were used for IP with anti-C protein MAb (produced in-mouse, Abmart) and immunoblotted with the anti-C protein (produced in-rabbit) and anti-PIAS4 polyclonal (AV33011, sigma) antibodies.

### Confocal imaging

Co-localization of C protein with PIAS4: BHK21 cells were co-transfected with pCDNA3.1-PIAS4-Myc and pCMV-HA plasmids as described previously and fixed in 4% paraformaldehyde for 30 min, permeated with 0.1% Triton X-100 for 15 min. The cells were blocked in 10% normal goat serum in PBS for 2 h. Next, cells were incubated for 4 h in the presence of rabbit anti-Myc PAbs and mouse anti-HA MAb, followed by a 1 h incubation in PBS containing anti-rabbit IgG (whole molecule)-fluorescein isothiocyanate (FITC) antibody (F9887; Sigma) and anti-mouse IgG -tetramethyl rhodamine isocyanate (TRITC) (whole molecule) antibody (T5393; Sigma). Nuclei were stained with 4, 6-diamidino-2-phenylindole (DAPI) for 15 min. The fluorescence signals were examined using a SP2 confocal fluorescence microscope (Leica).

Co-localization BVDV C protein with endogenous PIAS4: Confocal fluorescence microscopy was used for observation of co-localization in BVDV infected MDBK cell as described above. Anti-C protein MAb and anti-PIAS4 PAbs were used as primary antibodies, and anti-rabbit IgG-FITC antibody and anti-mouse IgG -TRITC were used as secondary antibodies.

### RNA interference

For the RNAi-mediated knockdown of PIAS4, GenScript (Nanjing) provided two different siRNAs against PIAS4. The target sequences of the si-PIAS4 were GGAGTAAGAGTGGACTTAAA (siPIAS4–1-1) and AGGCACTGGTCAAAGAGAA (siPIAS4–1-2). MDBK cells grown to 80% confluence in 6-well plates were transfected with 400 nM PIAS4 and scramble siRNA using X-treme GENE siRNA Transfection Reagent (Roche, Germany) as described [15]. At 48 hpt, the transfected cells were collected to detect cellular expression of PIAS4.

### Western blot analysis

HEK293T and MDBK cells were lysed with lysis buffer. After centrifugation, protein samples were separated by SDS-PAGE and transferred to a 0.2 μm nitrocellulose membrane (Hybond-C Super; GE Healthcare). Membranes were blocked at 4 °C overnight in block reagent (5% milk in PBS-Tween). For immunodetection, mouse anti-Myc MAb (1:1000), mouse anti-HA MAb (1:1000), mouse anti-beta MAb or rabbit anti-beta actin PAb (1:1000) (E021020, Earthox), rabbit anti-C protein PAbs (1:200), rabbit anti-PIAS4 MAb (1:1000), rabbit anti-Bax (1:1000) (CST, USA), rabbit anti-Bcl 2 (1:1000) (CST, USA), rabbit anti-STAT4 (1:1000) (CST, USA), rabbit anti-IFITM3(1:1000) (CST, USA), rabbit anti-SOCS3 (1:1000) (CST, USA), mouse anti-IFN α (1:1000) (CST, USA), rabbit anti-ISG15(1:1000) (CST, USA), rabbit anti-MX1(1:1000) (CST, USA) and rabbit anti-OAS1(1:1000) (CST, USA) were used as respective primary antibodies. Bound primary antibodies were detected by horseradish peroxidase-conjugated anti-mouse (Earthox), anti-rabbit (Earthox) antibodies. Immunoreactive bands were visualized using ECL kits.

### qRT-PCR

Cells were transfected with overexpression vector of core and PIAS4 genes. After transfection, cells were collected by centrifugation. The TRIzol method was used to isolate the RNA of virus and cells. The relative expression of different gene was defined as F = 2^-ΔΔct^.

### Statistical analysis

All the data were expressed as mean ± standard deviations. Student’s *t* test was used to compare two groups. Multiple groups were compared by one-way analysis (ANOVA) of variance and a *P* value of < 0.05 was considered significant (*P < 0.05, **P < 0.01).

## Results

### Identification of bovine PIAS4 as an interacting partner of BVDV C protein

**Fig. 1:**
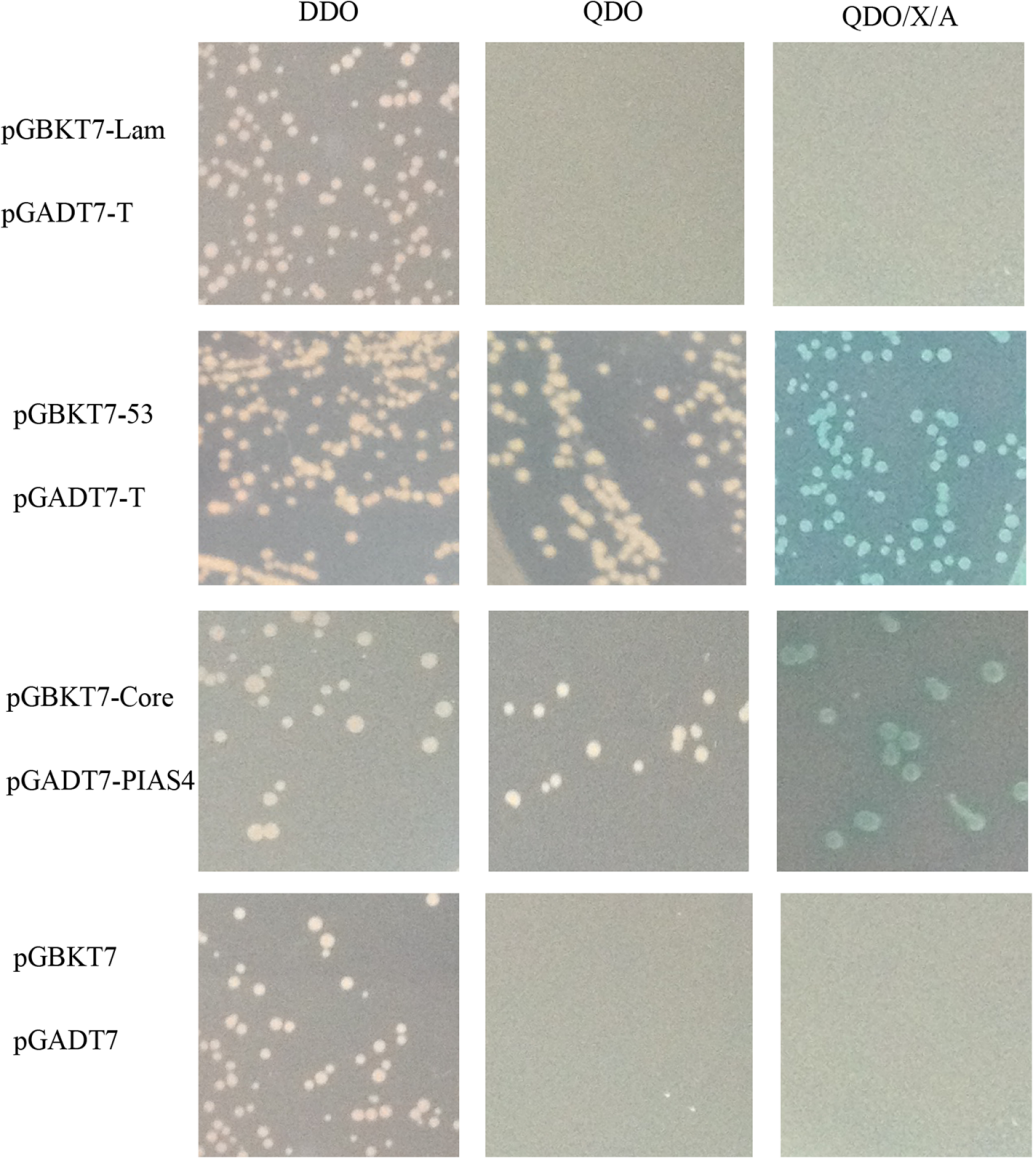
Reactivity of BVDV C protein with PIAS4 protein in a yeast two-hybrid system. Yeast strain Y2H was transformed with a bait plasmid, pGBKT7-Core, and Yeast strain Y187 was transformed with a prey plasmid, pGADT7-PIAS4. pGBKT7–53/pGADT7-T and pGBKT7-lam/pGADT7-T was used as positive and negative controls, respectively

To investigate the host interacting partners of BVDV C protein, we performed a yeast two-hybrid screening of a PBMC cDNA library using full-length C protein as bait. Positive colonies were selected for growth on QDO/X/A medium, and the plasmids were isolated and sequenced. In-frame proteins were retested for specificity to BVDV C protein. Thirteen proteins were identified as positive binding partners to BVDV C protein (Table 2). One of these proteins, PIAS4, was selected for further study because it has been shown previously to be involved in the antiviral immune response, and the interaction between BVDV C and PIAS4 was proved by yeast two-hybrid screening and indicated that was indeed specific (Fig. 1).

**Table 2:** Interactors screened by yest two-hybrid screen

Prey	No. of Genbank accession	No. of hits	similarity
*Bos taurus* small glutamine-rich tetratricopeptide repeat (TPR)-containing, alpha (SGTA)	NM_001038030	15	89%
Bos taurus protein inhibitor of activated STAT, 4	BC123606	13	93%
Bovine natural resistance-associated macrophage protein (Nramp)	NM_174652	5	92%
Bos taurus calcium modulating ligand (CAMLG)	NM_001037625	6	93%
Bos taurus cysteine-rich with EGF-like domains 1 (CRELD1)	NM_001014851	10	92%
Bos taurus transmembrane emp24-like trafficking protein (TMED10)	NM_001040549.2	4	92%
Bos taurus ribosomal protein L6 (RPL6)	BT021544.1	7	90%
Bos taurus tetratricopeptide repeat domain (TTC1)	NM_001034217.2	3	93%
Bos taurus reticulon-4 (RNT4), transcript variant 1	NM_001075138.1	5	93%
Bos taurus hypothetical protein LOC617407	BC108156.1	10	88%
Predicted:Ovis aries vascular cell adhesion protein 1-like,transcript variant 3	XM_004002235.1	5	89%
Bos taurus eukaryotic translation initiation factor 3, subunit E (EIF3E)	NM_001034603.1	2	80%
Bos taurus protein-L-isoaspartate (D-aspartate) O-methyltransferase	BC109663.1	2	93%

### Verification of the interaction between C protein and PIAS4 by co-Immunoprecipitation assay

**Fig. 2:**
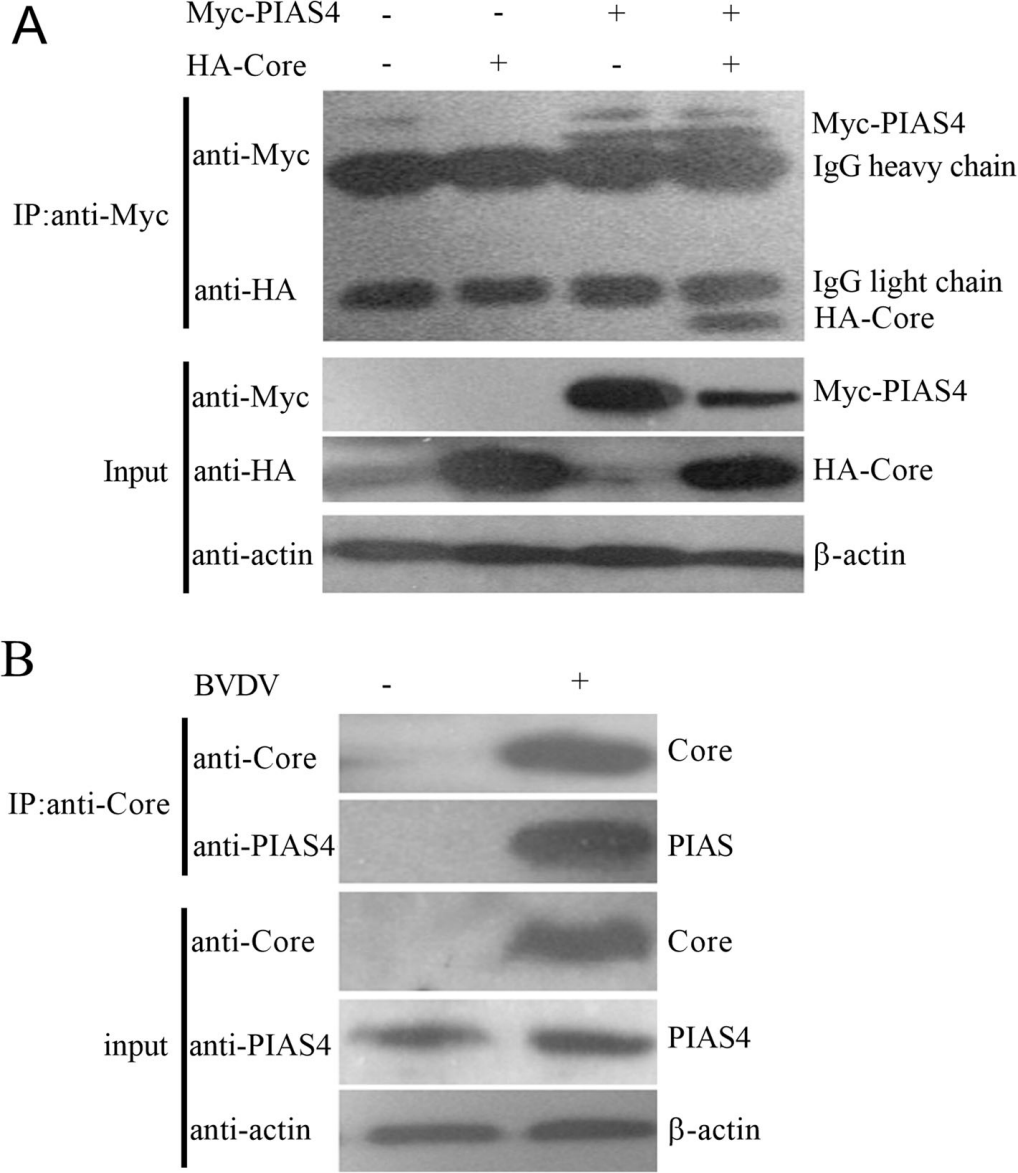
Verification the interaction of PIAS4 and C protein through Co-immunoprecipitation. **a** Co-IP of PIAS4 with core. The indicated plasmids were transfected into HEK293T cell, and whole cell lysates obtained 48 hpt were immunoprecipitated with rabbit anti-Myc PAbs; Proteins were detected by western blot (WB) with mouse anti-Myc and anti-HA MAb. **b** Co-IP of BVDV core with endogenous PIAS4. BVDV-infected or mock-infected MDBK cells were used for IP with anti-C protein MAb and immunoblotted with the anti-C protein and anti-PIAS4 PAbs

To confirm the interaction between C protein and PIAS4, co-IP assays were performed. The Myc-tagged PIAS4 and HA-tagged C protein were co-expressed in HEK 293 T cells. An antibody against Myc was used to co-IP from cell extracts. As shown in Fig. 2a, HA-C protein could be pulled down by the Myc antibody from cells co-expressing Myc-PIAS4 and HA-C protein but not from cells transfected single plasmid. To test the association between C protein of BVDV and cellular PIAS4 in MDBK cells, BVDV-infected cells lysates were immuno-precipitated with anti-C protein MAb and probed for the presence of PIAS4. Uninfected cells were used as a negative control. The results showed that C protein is able to bind to PIAS4 in MDBK cell upon infection with BVDV (Fig. 2b).

### Analysis of co-localization between C protein and PIAS4 by confocal microscopy

**Fig. 3:**
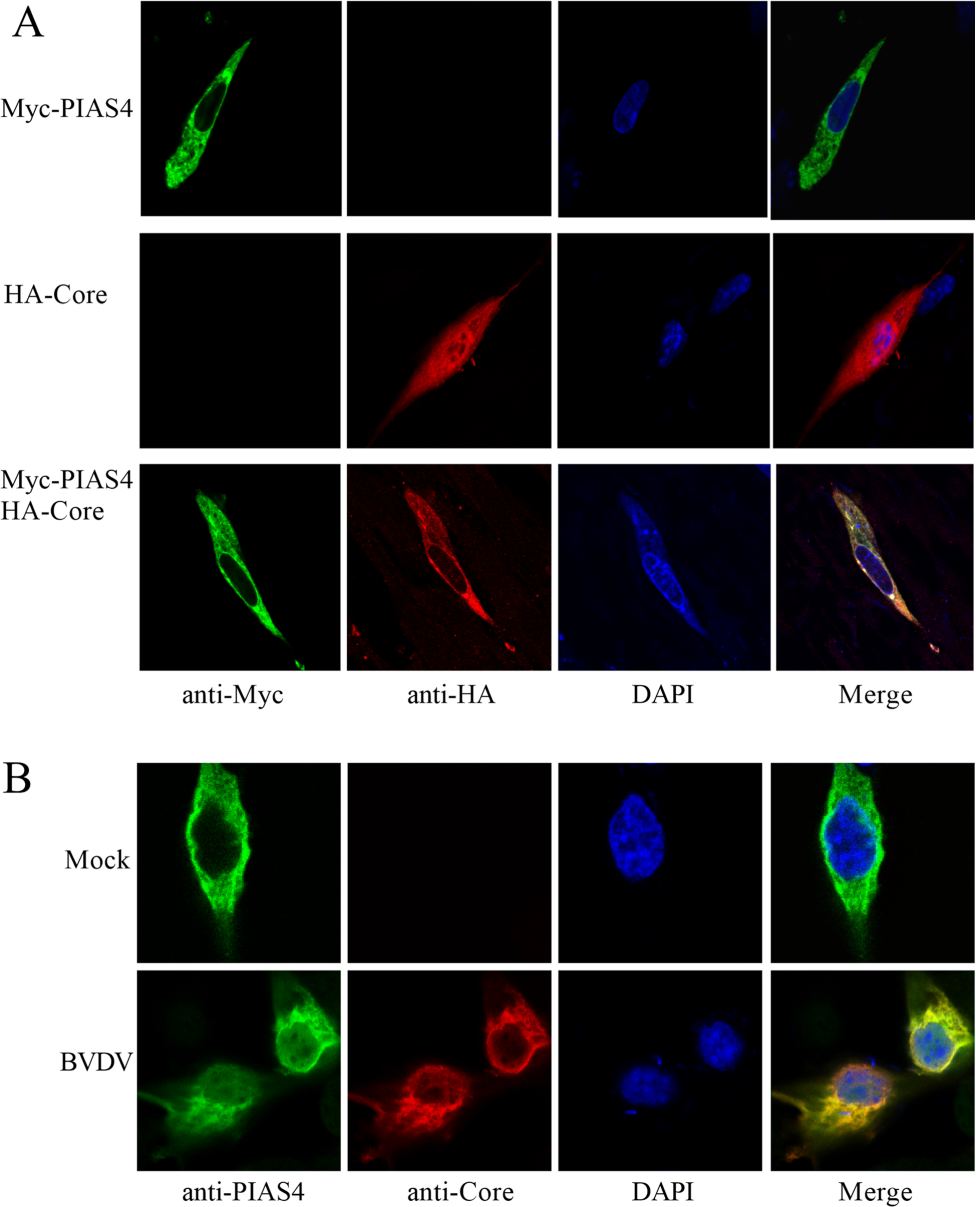
The co-localization between protein and PIAS4 by confocal microscopy. **a** Co-localization of core with PIAS4. BHK21 cells were transfected with pCDNA3.1-PIAS4-Myc and pCMV-HA-C or plasmids with switched tags, and subjected to indirect immunofluorescence to detect tagged C protein and PIAS4 using rabbit anti-Myc PAbs and mouse anti-HA MAb. **b** Co-localization of BVDV core with endogenous PIAS4. MDBK cells were infected with the BVDV and then subjected to indirect immunofluorescence to detect C protein and PIAS4 using anti-C protein MAb and anti-PIAS4 PAbs

To further examine co-localization of C protein with PIAS4, BHK21 cells were co-transfected by the plasmids pCDNA3.1-PIAS4-Myc and pCMV-HA-C protein, and the subcellular localization of C protein and PIAS4 was verified by confocal microscopy. The results indicated that both Myc-PIAS4 and HA-C were distributed throughout the cytoplasm. Cells co-transfected with empty vectors pCDNA3.1/myc-His(−)A and PMC-HA-C, or empty vectors PMC-HA and pCDNA3.1-PIAS4-Myc were used as control, which showed no interaction between Myc and HA (Fig. 3a). To confirm that endogenous PIAS4 co-localizes with C protein, MDBK cells were mock-infected or infected BVDV. Staining with anti-PIAS4 PAbs and anti-C MAb showed that C protein co-localized with PIAS4 in the cytoplasm (Fig. 3b).

### Effect of PIAS4 expression on BVDV growth and C protein expression

**Fig. 4:**
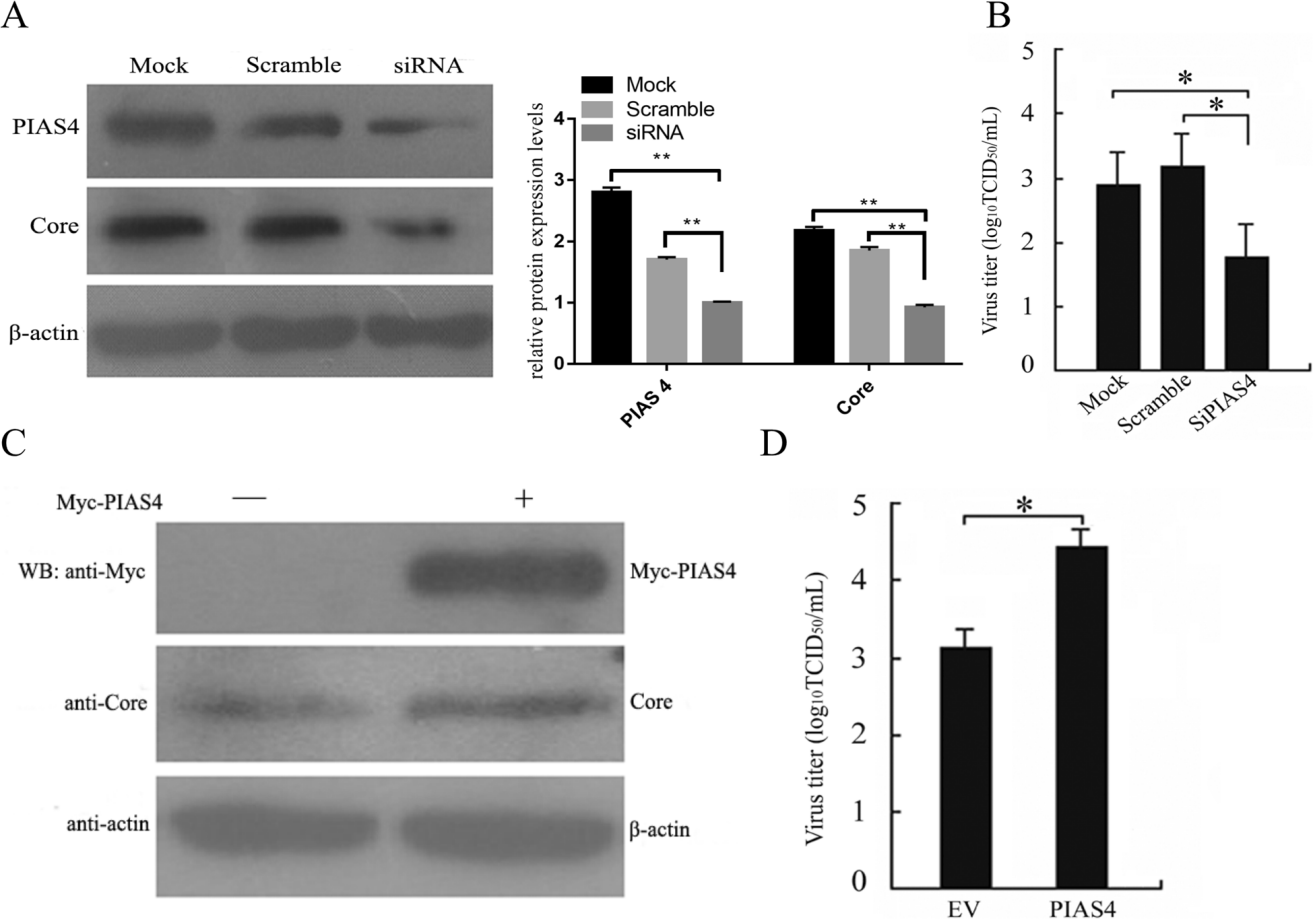
The effect of PIAS4 expression to C protein expression and viral titer. **a** Knockdown of PIAS4 protein levels by siRNA treatment. MDBK cells were transfected with no siRNA (Mock), scramble siRNA (siScr), siRNA targeting PIAS4 and harvested at 48 hpt. The PIAS4 and C protein were detected by immunoblotting. **b** BVDV titers in PIAS4 knockdown cells. **c** Increased C protein expression in PIAS4-overexpressing cells. MDBK cells were transfected with pCDNA3.1-myc-PIAS4 (PIAS4) and empty vector (EV) for 12 h, followed by infection with BVDV for 48 h. The expression levels of C-protein and PIAS4 were analyzed by WB. **d** Increased BVDV growth in PIAS4-overexpressing cells. The detection of virus titers was described above. The virus titers in the supernatants of all treatment groups were determined by virus titration assays and expressed as TCID_50_/0.1 ml. Error bars represent the standard error of the mean from three independent experiments. *P < 0.05, **P < 0.01 were considered statistically significant

To test whether C-PIAS4 interaction was capable of influencing BVD viral replication, MDBK cells were transfected with siRNA specific to PIAS4, which exhibited an obvious decrease of endogenous PIAS4 and viral C protein expression levels compared to that cells treated with a scramble siRNA (siScr) and mock-treated cell (Fig. 4a). In different treatments, the difference of PIAS4 and C protein expression was significant (*P* < 0.01). In addition, inhibited PIAS4 protein level resulted in a reduction in virus titer in cell supernatant (Fig. 4b). The results indicated that virus growth and C protein expression were decreased after inhibition of cellular PIAS4.

The inhibitory effects of PIAS4 deletion on viral growth and protein expression prompted investigation of the implication of PIAS4 overexpression on BVDV growth and C protein expression. To this end, MDBK cells were transfected with plasmid pCDNA3.1-PIAS4-Myc, followed by BVDV infection. As shown in Fig. 4c, BVDV C protein expression was increased when PIAS4 was upregulated as compared to empty vector (EV) transfected cell. Similarly, an increase in BVD viral titer was detected when PIAS4 was overexpressed in MDBK cells (Fig. 4d). These results together showed that PIAS4 is required for effective infection of BVDV.

### The effect of PIAS4 and C protein of BVDV on host antiviral responses

**Fig. 5:**
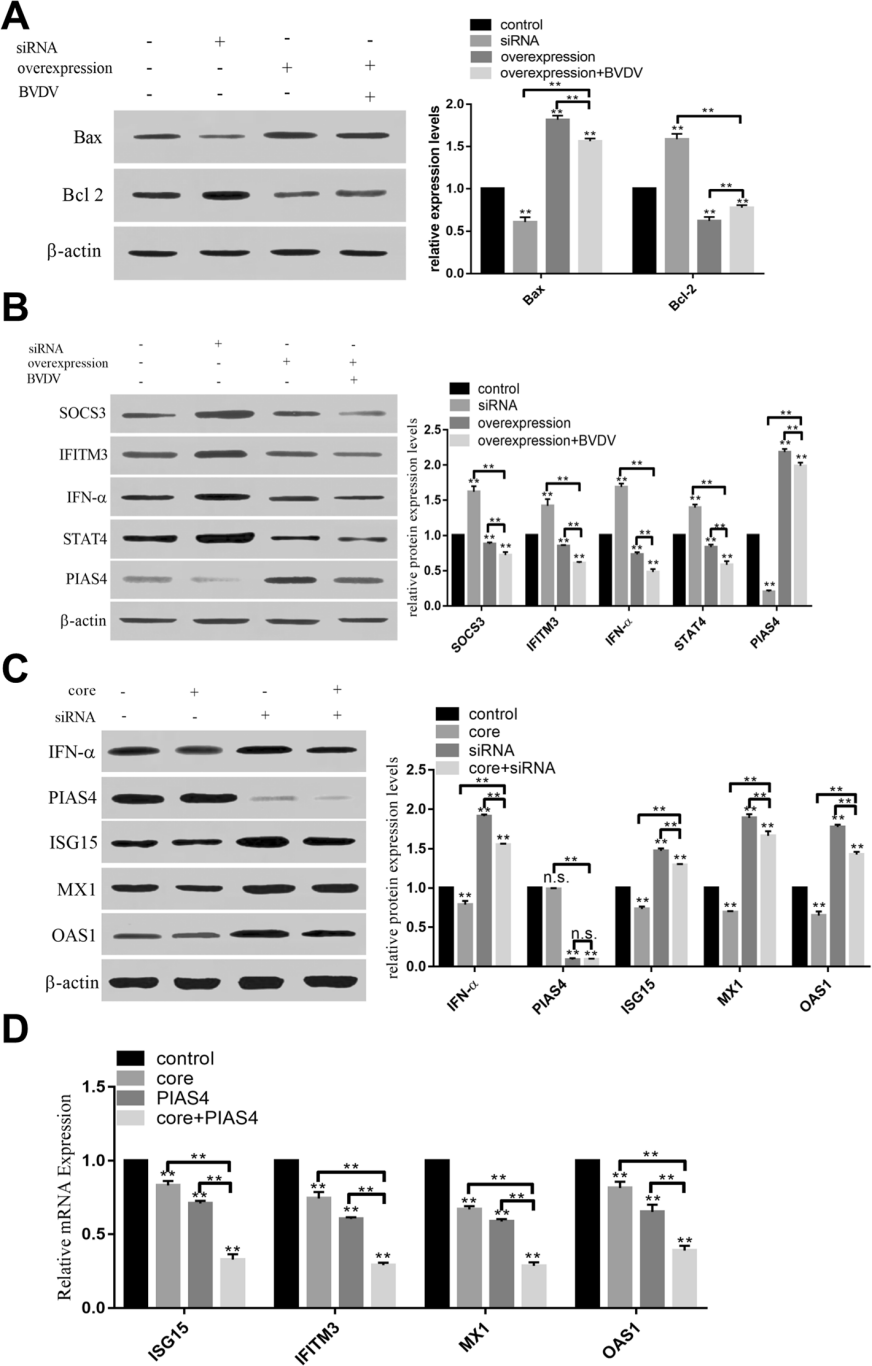
The effect of PIAS4 expression on immunity response. **a** To determine the effect of PIAS4 expression on host cell survival. The expression of BAX and Bcl-2 was analyzed by western blot under the conditions that PIAS4 expression was changed and the cells were infected by BVDV. **b** Overexpression of PIAS4 inhibited the expression of SOCS3, IFITM3 IFN-ɑ and STAT4 in protein levels. **c** The expression of core protein downregulated antivirus protein expression when cells were transfected with siRNA (PIAS4). d The expression of ISG15, IFITM3, MX1, OAS1 was downregulated by interaction of PIAS4 and core protein

To determine the effect of PIAS4 on host antiviral immunity, overexpression and siRNA inhibition of PIAS4 were carried out. Results showed that overexpression of PIAS4 promoted apoptosis of host cells, increased the expression of Bax, and decreased the expression of Bcl-2 (Fig. 5a). Whereas, the expression of PIAS4 inhibited the expression of SOCS3, IFITM3 and IFNα and STAT 4 signal. And the expression of SOCS3, IFITM3, IFN-ɑ, STAT4 was further inhibited in cells overexpressing PIAS4 and infecting by BVDV (Fig. 5b). Overexpression of C protein downregulated the expression of IFN-α, ISG15, MX1 and OAS1 in cells expressing PIAS4 or lacking PIAS4 (Fig. 5c-d).

## Discussion

The PIAS family consists of PIAS1, PIAS2, PIAS3 and PIAS4 which are initially identified as negative regulators of STAT signaling [16, 17]. There are four main mechanisms by which PIAS proteins negatively regulate transcription [18]. Frist, a PIAS protein might block the DNA-binding activity of a transcription; second, PIAS proteins might recruit other coregulators; third, a PIAS protein might repress transcription by sumoylation of a transcription factor, and the last, PIAS proteins might repress transcription by sequestering transcription factors. For example, PIAS4 is a transcriptional corepressor of STAT1, which represses STAT-1 mediated gene activation [19]. PIAS4 could interact with histone deacetylases (HDACs) and the ability of PIAS4 to repress the transcriptional activity of SMAD3 [20]. PIAS4 overexpression inhibited the TNF-β mediated signaling [20]. With the extensively progress for PIAS4 related researches, PIAS4 protein regulated immune responses and other cellular functions through the modulation of transcription factors, such as, the repression of IRF3, IRF7, TRIF, LEF-1, MYB-mediation transcription [21, 22]. In present study, we employed a yeast two-hybrid assay to find PIAS4 that interact with the BVDV C protein and then used to confirm their interaction. By confocal analysis, PIAS4 and C protein were found to interact and co-localize in the cytoplasm. When expressed ectopically, PIAS4 increased BVDV replication in infected MDBK cells, the virus titer was significantly higher compared to empty vector transfection (*P* < 0.05). Consistent with previous study, PIAS4 can enhance the replication of RNA virus [8].

In our study, we determined that the binding of BVDV C protein to PIAS4 can enhance viral replication and proliferation. We speculated that such effect may be related to the negative regulation of type I interferon (IFN) transcription by PIAS4. The interaction of PIAS4 and core protein deepened the inhibitory effect of antivirus protein expression, and facilitated the accumulation of virus RNA [23]. This is consistent with our results. Moreover, cytokines have been shown to enhance the immune escape of BVDV when PIAS4 was overexpressed [24]. And antagonizing JAK-STAT signaling pathway is one of the strategies of viruses involves in resisting the host antiviral immune responses [25].

Besides, PIAS4 relied on the SUMO modification mechanism to inhibit IFN transcription as knockdown of SUMO E2 enzyme UBC9 decreased inhibitory activity of PIAS4. In case of CSFV, SUMOylation can affect viral infection, and SUMOylation of C proteins is associated with the pathogenesis and toxicity of CSFV. The C protein SUMOylation-mutant CSFV has severe replication defects during animal infection [8]. Indeed, most interaction protein partners of PIAS proteins can be modified by SUMO [26]. PIAS proteins can act as SUMO E3 ligases in the enzymatic reaction, which attaches small ubiquitin-related modifier to target proteins [27–29]. SUMOylation has been reported to regulate several biological processes, it can alter the activity, stability, or subcellular localization of its targets [30]. In CSFV, the C protein interacts with UBC9 and SUMO-1 in the SUMOylatoin pathway, affecting the virulence of the virus [31]. Whether PIAS4-dependent SUMOylation involved in BVDV virulence needs to be further studied in the future research.

## Conclusion

In conclusion, we identified cellular PIAS4 as a novel interacting partner of BVDV C protein. PIAS4 positively regulates replication and growth of BVDV through the regulation of host cell apoptosis and innate immune responses.

## Data Availability

The datasets in this study are available from the corresponding author on reasonable request.

## Abbreviations

BVDV: Bovine viral diarrhea virus
CSFV: Classical swine fever virus
STAT: Signal transducer and activator of transcription
Co-IP: Co-immunoprecipitation
HDACs: histone deacetylases
IFN: Interferon
PIAS: Protein inhibitor activated STAT
SUMO: Small ubiquitin-related modifier
ORF: Open reading frame
PBMC: Peripheral blood mononuclear cell
SOCS: Suppressor of cytokine signaling
JAK: Janus kinase
MDBK: Madin-darby bovine kidney
WB: Western blot
